# Efficacy of Nanocurcumin as an Add-On Treatment for Patients Hospitalized with COVID-19: A Double-Blind, Randomized Clinical Trial

**DOI:** 10.1155/2023/5734675

**Published:** 2023-07-28

**Authors:** Sedigheh Ahmadi, Zeinab Mehrabi, Morteza Zare, Sara Ghadir, Seyed Jalil Masoumi

**Affiliations:** ^1^Student Research Committee, Shiraz University of Medical Sciences, Shiraz, Iran; ^2^Department of Internal Medicine, Shiraz University of Medical Sciences, Shiraz, Iran; ^3^Nutrition Research Center, School of Nutrition and Food Sciences, Shiraz University of Medical Sciences, Shiraz, Iran; ^4^Gastroenterohepatology Research Center, Shiraz University of Medical Sciences, Shiraz, Iran; ^5^Center for Cohort Study of SUMS Employees' Health, Shiraz University of Medical Sciences, Shiraz, Iran

## Abstract

**Background:**

Curcumin is a polyphenol derivative of the *Curcuma longa* rhizome, with potential antioxidant, anticancer, antidepressant, antiviral, and anti-inflammatory effects. This compound can be prepared as biodegradable polymer nanoparticles, called nanocurcumin, to improve its solubility, stability, half-life, and bioavailability.

**Aim:**

We explored nanocurcumin's effect on the clinical manifestations of patients hospitalized with mild-to-moderate COVID-19.

**Methods:**

This double-blind, randomized clinical trial involved 76 COVID-19 patients admitted to Ali-Asghar Hospital from December 2021 to March 2022. All patients received standard coronavirus treatment as per national guidelines. In addition, four times a day for two weeks, the curcumin group received 40 mg of nanocurcumin, while the control group received a placebo. Clinical manifestations were examined and recorded by the associate doctors working in the department. Statistical analysis was done using SPSS v. 21.

**Results:**

Thirty-nine people from the control group and 29 from the curcumin group completed the study. At baseline, the groups were comparable in age, gender, body mass index, hospitalization duration, and background diseases. The mean age of patients in the control and treatment groups was 53.9 ± 11.9 and 54.6 ± 13.4, respectively. Compared with the placebo, nanocurcumin minimized coughs (*P*=0.036), fatigue (*P*=0.0001), myalgia (*P*=0.027), oxygen demand (*P*=0.036), oxygen usage (*P*=0.05), and respiratory rate (*P* < 0.0001). By discharge, the curcumin group had a significantly greater increase in SPO_2_ than the control group (*P*=0.006).

**Conclusions:**

This preliminary study suggests that nanocurcumin has a potentiating anti-inflammatory effect when combined with standard COVID-19 treatment, helping the recovery from the acute inflammatory phase of the disease in hospitalized patients with mild-to-moderate disease severity. This trial is registered with Iranian Registry of Clinical Trials: IRCT20211126053183N1 (registered while recruiting on 13/12/2021).

## 1. Introduction

The coronavirus disease 2019 (COVID-19) pandemic has affected roughly 567 million people to date [1]. Presentations vary from mild flu-like symptoms to acute respiratory distress syndrome and death. Fever, fatigue, and cough are the most common symptoms among patients with mild COVID-19. In contrast, chest pain, difficulty in breathing, and hypoxia are common symptoms in moderate and severe cases. The disease may cause acute respiratory distress syndrome (ARDS), total organ failure, and death [2]. The immune system response causes a hazardous phenomenon called cytokine storms by producing plentiful amounts of inflammatory cytokines [3]. This cytokine release syndrome accounts for acute inflammation, coagulopathies, a hyper-immune response, and thromboembolic events, leading to dangerous outcomes in COVID-19 patients [4, 5].

Curcumin is a polyphenol derivative of the *Curcuma longa* rhizome [6]. Around 3,000 preclinical studies have been conducted, and many have reported curcumin's potential beneficial effects and safety (tolerated up to 12 g/day) [7]. Moreover, some studies have reported antioxidant [8], anticancer [9], and antidepressant [10] effects of curcumin. In several in vivo and in vitro studies, this traditional herb significantly inhibited the generation and secretion of proinflammatory cytokines like IL-1, IL-6, IL-8, and TNF-*α* [11, 12]. Additionally, several animal studies have reported that curcumin reduces IL-1b, IL-6, and TNF-*α* levels in infectious lung injuries [11, 12]. Curcumin can also diminish the production of several cytokines and chemokines, such as the MMP family, MCP1, MIPI1, SDF1, and the CXCL family [13, 14]. Curcumin also has antiviral effects against different viruses [15–18]. Zahedipour et al. reported that curcumin has potential antiviral effects against severe acute respiratory syndrome coronavirus 2 (SARS-CoV-2) through inflammatory modulation and/or immunological responses, viral inhibition, and the ability to reverse the pulmonary edema and fibrosis-associated pathways in COVID-19 [19]. Furthermore, curcumin can be prepared as biodegradable polymer nanoparticles (“nanocurcumin”) to improve its solubility, stability, half-life, and bioavailability [20].

Considering the cytokine storm and inflammatory state caused by SARS-CoV-2, we conducted a randomized clinical trial to assess the effect of nanocurcumin on the clinical manifestations of patients hospitalized with COVID-19.

## 2. Methods

### 2.1. Study Design

This double-blind, randomized clinical trial was conducted at Ali-Asghar Hospital in Shiraz, Iran, from December 2021 to March 2022. Seventy-six COVID-19 patients admitted to this hospital were enrolled. Randomization was done using a table of random numbers.

### 2.2. Patients

**Figure 1:**
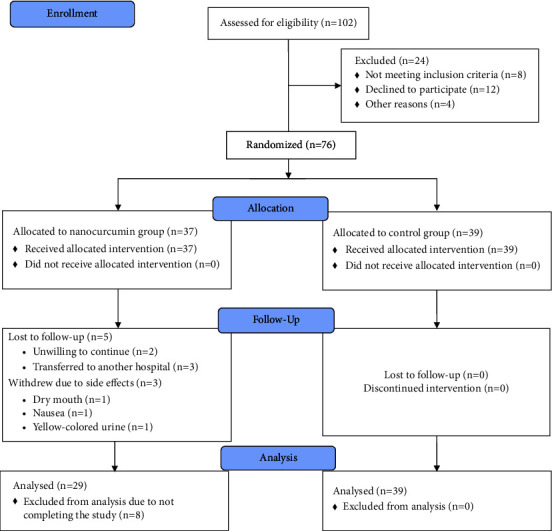
CONSORT flow diagram of the study.

This study included hospitalized COVID-19 patients aged 18 to 80 with a positive RT-PCR for COVID-19 or pulmonary involvement on imaging. Participation was voluntary. A total of 102 patients were assessed for eligibility (Figure 1). Excluded were those with a history of an allergic reaction to curcumin, those unable to use more than a quarter of the curcumin supplements, those who were intubated, those who had severe renal failure (GFR < 30) or needed hemodialysis, those with active gastrointestinal bleeding, malignancies, cholecystitis, hemophilia, or other coagulopathies, those who were pregnant or breastfeeding, and those participating in other clinical studies.

### 2.3. Sample Size

In line with a previous study [21], the sample size was calculated using the sample size formula for interventional studies considering *α* = 0.05, 80% test power, and a 20% dropout rate. Accordingly, our target was roughly 38 individuals in each group.

### 2.4. Intervention

During hospitalization, the patients of both groups received standard coronavirus treatment according to the national protocol for the treatment of COVID-19 [22]. This included remdesivir, anticoagulants (heparin or enoxaparin), dexamethasone, zinc sulfate supplements (10 mg of elemental zinc), and vitamin B complex tablets (B1 5 mg, B2 2 mg, and B6 2 mg). They also received 500 mg of vitamin C on all days of hospitalization and a single dose of 50,000 units of vitamin D on the first day.

This study trialed curcumin nanomicelles manufactured by Exir Nano Sina Company (Iran), referred to as “nanocurcumin.” These biodegradable polymer nanoparticles can improve curcumin's solubility, stability, half-life, and bioavailability [20]. Patients in the curcumin group were treated with one Sina-curcumin capsule (containing 40 mg of nanocurcumin) four times daily (morning, noon, afternoon, and before sleep) for two weeks (i.e., a total of 160 mg daily). For patients in the control group, the same company prepared a placebo of precisely the same shape and color, administered in the same way. The placebo contained gelatin and soybean oil (also present in the Sina-curcumin formula) but did not include the active nanocurcumin substance. In this double-blind study, the physicians who prescribed the drug and the patients who participated in the project did not know who received a placebo or the main supplement containing nanocurcumin.

### 2.5. Outcomes

A clinical examination questionnaire was filled out for hospitalized patients on the first and fourteenth days of the study. If the patients were discharged, they would be asked to return to the hospital's clinic for evaluation. Anthropometric criteria, including weight and height, were taken from the patients at the beginning of the study. Clinical symptoms including cough, muscle pain, sore throat, diarrhea, nausea, vomiting, change in the sense of taste and smell, fatigue, chills, confusion, feeling pain or pressure in the chest, number of breaths per minute, need for oxygen mask, and oxygen saturation level (SPO_2_) were assessed. Clinical examinations were performed by the physicians working in the department, and the related tests were done in the hospital or approved laboratories of Shiraz University of Medical Sciences.

### 2.6. Statistical Analysis

SPSS version 21 was used to analyze the collected data. Mean and standard deviation and frequency and frequency percentage were used to describe quantitative and qualitative variables, respectively. The Shapiro–Wilk test was used to measure the normality of the data. To calculate the mean differences before and after the intervention in each group, the paired *t*-test and the Wilcoxon test were used for variables with and without normal distribution, respectively. To calculate the mean differences between the two groups, the *t*-test and Mann–Whitney test were used for the variables with and without normal distribution, respectively. *P* values below 0.05 were considered significant in all cases.

### 2.7. Ethical Considerations

According to the entry and exit criteria of the study, the supplement was found to be safe and entirely ethical for people eligible to enter the present study. In addition, at the beginning of the study, the subject, objectives, and method of the study were explained to the patients. Then, if they were willing to participate, a written informed consent form was completed. All participants were allowed to withdraw at any time and at any stage of the research. We followed the principles outlined by the Declaration of Helsinki at all stages. The study proposal was approved by the Ethics Committee of Shiraz University of Medical Sciences under the code IR.SUMS.SCHEANUT.REC.1400.031. It is also registered with the Iranian Registry of Clinical Trials (IRCT20211126053183N1).

## 3. Results

Of the 76 people who were willing to enter the study and met the inclusion criteria, three people in the curcumin group dropped out due to unwillingness to continue, two due to being transferred to another hospital, and three withdrew due to side effects (one with dry mouth, one with nausea, and one with yellow-colored urine). Finally, 39 people in the placebo group and 29 people in the curcumin group completed the study (Figure 1).

In this study, the average age of patients in the control and treatment groups was 53.9 ± 11.9 and 54.6 ± 13.4, respectively. At baseline, the study groups were comparable in terms of age, gender, body mass index, hospitalization duration, and background diseases such as diabetes, hypertension, dyslipidemia, cardiovascular diseases, and rheumatoid arthritis (Table 1). In the control group, 24 (61.5%) patients were male, and 15 (38.5%) were female. In the treatment group, 11 (37%) and 18 (62%) patients were male and female, respectively.

As shown in Table 2, there were no significant differences between the two groups in clinical parameters (*P* > 0.05). By the day of discharge, although the rate of cough was significantly reduced in both groups, the nanocurcumin supplement induced a significant difference between the groups in this parameter (*P*=0.036). Nanocurcumin did not induce any difference between the two groups in sore throat (*P*=0.216) and nausea (*P*=0.243). Additionally, the supplement reduced the symptoms of fatigue (*P*=0.0001) and myalgia (*P*=0.027). Nanocurcumin also reduced the oxygen demand (*P*=0.036), the hours of oxygen usage (*P*=0.05), and respiratory rates (*P* < 0.0001). By the day of discharge, the curcumin group had a significantly greater increase in SPO_2_ than the control group (*P*=0.006). Comparing the control and treatment groups, curcumin was not effective on diarrhea (*P*=0.379), anorexia (*P*=0.901), chills (*P*=0.831), chest pain (*P*=0.26), anosmia (*P*=0.695), ageusia (*P*=0.776), and weight (*P*=0.84).

## 4. Discussion

In the present study, we investigated the therapeutic role of curcumin nanomicelles as a mediator of inflammatory immune responses in COVID-19 patients. Our study shows that nanocurcumin can be an effective anti-inflammatory drug for treating patients with COVID-19. Nanocurcumin significantly reduced the prevalence of cough, fatigue, and myalgia caused by COVID-19 compared with the control group. Moreover, this supplement reduced oxygen demand and oxygen mask usage. The respiratory rates were significantly declined by nanocurcumin. After two weeks, the oxygen saturation of patients in the treatment group was significantly higher than those in the control group.

The COVID-19 pandemic, which emerged in late 2019 and spread worldwide, is still a global concern. Despite many efforts to find a cure, no definite drug or treatment has been found. It is known that the severe form of COVID-19 causing extended lung damage and mortality is a result of cytokine storm and extreme immune response [23]. Our clinical trial highlights the anti-inflammatory effects of curcumin. Similar to our results, in a clinical trial performed by Saber-Moghaddam et al., curcumin could significantly improve clinical outcomes, including length of hospital stay and blood oxygen level, compared with the placebo group [24]. A triple-blind, placebo-controlled, randomized clinical trial reported that nanocurcumin administration in the inflammatory phase of COVID-19 modulates inflammatory immune responses and therefore accelerates recovery [25].

The anti-inflammatory effects of curcumin have been studied before. Curcumin is one of the most potent anti-inflammatory agents [26]. Saadati et al. reported that curcumin reduced fibrosis, nuclear factor-kappa B activity, TNF-*α* serum level, and liver enzymes in non-alcoholic fatty liver patients [27]. In another study on type 2 diabetes patients, curcumin improved the serum lipid profile, CRP, and adiponectin levels [28]. Some studies have reported curcumin's therapeutic impact on Th17 cell-mediated diseases like multiple sclerosis [29], rheumatoid arthritis [30], and Alzheimer's disease [31]. Tahmasebi et al. showed that in the curcumin group in both mild and severe COVID-19 patients, the Th17 cell numbers, Th17 cell-related factors, and Th17 cell-related cytokines were reduced [32]. In another study by Tahmasebi et al., curcumin led to alterations of anti-inflammatory factors, including increased frequency of suppressor T-reg cells, increased secretion of anti-inflammatory cytokines, and elevated levels of FOXP3, IL10, IL35, and TGF-*β* in COVID-19 patients [33].

Curcumin causes this immunomodulation via several mechanisms. It inhibits IKKb activation and increases IKBa production [11, 34, 35]. It also activates AMPK and prevents nuclear translocation of NF-kB and *P*65, which finally reduces inflammation [36, 37]. Additionally, curcumin affects cyclooxygenase 2 (COX-2) activity [38]. Curcumin can also act on the cellular immune system as it inhibits the migration and accumulation of main immune cells responsible for cellular immune responses such as CD4+ and CD8+ T lymphocytes, natural killer cells, and peripheral mononuclear cells (PMN cells) [39]. So, curcumin is able to impact immune response.

The application of pure curcumin is not practical and cost-effective because of its rapid metabolism, low bioavailability, weak aqueous solubility, and systemic deletion [40]. Hence, nanocurcumin (biodegradable polymer nanoparticles) was manufactured to improve curcumin's solubility, stability, half-life, and bioavailability [20]. Our study used this agent and found promising results in patients hospitalized with mild-to-moderate COVID-19.

Our study faced certain limitations. Firstly, the dropout rate in the curcumin group was relatively high, meaning that factors that prevent adherence to this therapy should be addressed. Secondly, vaccination status was not considered, and the results may not be generalizable to different populations and SARS-CoV-2 strains. However, as over half of Iran's population had received at least one COVID-19 vaccine before the start of the study [41] and considering the randomized design, it can be assumed that any effect of vaccination would be equal across the two groups. Hence, this trial's double-blinded, randomized, and controlled design renders its results valid and valuable as a preliminary study. Further double-blinded large sample-sized trials in different populations are needed to confirm our findings; nanocurcumin should also be evaluated regarding a possible prophylactic role in the fight against COVID-19.

## 5. Conclusion

This double-blind, randomized clinical trial suggests that prescribing nanomicelles containing curcumin to patients hospitalized with mild-to-moderate COVID-19 can improve inflammatory symptoms such as cough, fatigue, and myalgia. Also, nanocurcumin reduced the demand and time of oxygen therapy and improved blood O_2_ saturation. Therefore, nanocurcumin appears to have a potentiating anti-inflammatory effect when combined with standard COVID-19 treatment.

## Figures and Tables

**Table 1:** The demographic and background features of patients.

Variables	Control	Treatment	*P* value
Age (years)	53.9 ± 11.9	54.6 ± 13.4	0.813^*∗*^
Gender			
Male	24 (61.5%)	11 (37%)	0.054^*∗∗*^
Female	15 (38.5%)	18 (63%)	0.887
Body mass index (kg/m^2^)	30.4 ± 12.3	27.86 ± 4.6	0.579^*∗*^
Hospitalization duration (days)	5.75 ± 3.27	5.40 ± 1.52	0.623^*∗*^
Diabetes mellitus	10 (25.6%)	7 (24.1%)	0.479^*∗∗*^
Hypertension	11 (28.2%)	6 (20.7%)	0.292^*∗∗*^
Dyslipidemia	11 (28.2%)	5 (17.2%)	0.611^*∗∗*^
Cardiovascular diseases	5 (12.8%)	5 (17.2%)	0.583^*∗∗*^
Rheumatoid arthritis	4 (10.3%)	2 (6.8%)	0.583^*∗∗*^

^*∗*^Independent *t*-test; ^*∗∗*^Pearson's chi-squared test.

**Table 2:** The effect of nanocurcumin on the symptoms and clinical features of patients hospitalized with COVID-19.

Variables	Control			Treatment			*P* value (day 0)	*P* value (discharge)
	Day 0	Day of discharge	*P* value	Day 0	Day of discharge	*P* value		
Cough	35 (89.7%)	16 (41%)	<0.001^*∗*^	27 (93.1%)	5 (17.2%)	<0.001^*∗*^	0.629^*∗∗*^	0.036^*∗∗*^
Sore throat	12 (30.8%)	2 (5.1%)	0.002^*∗*^	12 (41.4%)	0	—	0.365^*∗∗*^	0.216^*∗∗*^
Nausea	6 (15.4%)	0	—	5 (17.2%)	1 (%3.4)	0.21^*∗*^	0.837^*∗∗*^	0.243^*∗∗*^
Fatigue	30 (76.9%)	26 (66.7%)	0.289^*∗*^	22 (75.9%)	7 (24.1%)	<0.001^*∗*^	0.919^*∗∗*^	0.0001^*∗∗*^
Myalgia	20 (51.3%)	6 (15.4%)	0.001^*∗*^	14 (48.3%)	0	—	0.806^*∗∗*^	0.027^*∗∗*^
Diarrhea	1 (2.6%)	1 (2.6%)	1^*∗*^	3 (10.3%)	0	—	0.177^*∗∗*^	0.379^*∗∗*^
Anorexia	26 (66.7%)	3 (7.7%)	<0.001^*∗*^	16 (55.2%)	2 (6.9%)	<0.001^*∗*^	0.335^*∗∗*^	0.901^*∗∗*^
Chills	15 (38.5%)	1 (2.6%)	<0.001^*∗*^	8 (27.6%)	1 (3.4%)	0.039^*∗*^	0.349^*∗∗*^	0.831^*∗∗*^
Chest pain	22 (56.4%)	8 (20.5%)	<0.001^*∗*^	18 (62.1%)	3 (10.3%)	0.001^*∗*^	0.639^*∗∗*^	0.26^*∗∗*^
Anosmia	21 (53.8%)	21 (53.8%)	1.00^*∗*^	19 (65.6%)	17 (58.6%)	0.62^*∗*^	0.333^*∗∗*^	0.695^*∗∗*^
Ageusia	18 (46.2%)	12 (30.8%)	0.031^*∗*^	16 (55.2%)	8 (27.6%)	0.021^*∗*^	0.462^*∗∗*^	0.776^*∗∗*^
Oxygen demand	33 (84.6%)	16 (41%)	<0.001^*∗*^	23 (79.3%)	5 (17.2%)	<0.001^*∗*^	0.57^*∗∗*^	0.036^*∗∗*^
Oxygen mask (hrs)	19.24 ± 6.8	3.3 ± 8.8	<0.001^*∗∗∗*^	15.93 ± 8.8	0	<0.001^*∗∗∗*^	0.63^*∗∗∗∗*^	0.053^*∗∗∗∗*^
Respiratory rate	21.2 ± 1.51	21.23 ± 1.6	0.809^*∗∗∗*^	20.5 ± 1.12	20 ± 0.617	0.012^*∗∗∗*^	0.187^*∗∗∗∗*^	<0.001^*∗∗∗∗*^
Oxygen saturation	89.84 ± 3.4	90.93 ± 3.26	0.002^*∗∗∗*^	89.93 ± 2.91	93.25 ± 2.5	<0.001^*∗∗∗*^	0.89^*∗∗∗∗*^	0.006^*∗∗∗∗*^
Weight	79.91 ± 16.2	79.81 ± 16.7	<0.001^*∗∗∗*^	79.43 ± 16.6	78.83 ± 16.6	<0.001^*∗∗∗*^	0.88^*∗∗∗∗*^	0.84^*∗∗∗∗*^

^*∗*^McNemar test; ^*∗∗*^Pearson's chi-squared test; ^*∗∗∗*^Wilcoxon signed-rank test; ^*∗∗∗∗*^Mann–Whitney test.

## Data Availability

The data used to support the findings of this study are available from the corresponding author upon reasonable request.
